# Pseudotyping Improves the Yield of Functional SARS-CoV-2 Virus-like Particles (VLPs) as Tools for Vaccine and Therapeutic Development

**DOI:** 10.3390/ijms241914622

**Published:** 2023-09-27

**Authors:** Andrew J. Zak, Trang Hoang, Christine M. Yee, Syed M. Rizvi, Ponnandy Prabhu, Fei Wen

**Affiliations:** Department of Chemical Engineering, University of Michigan, Ann Arbor, MI 48109, USAprabhup@umich.edu (P.P.)

**Keywords:** SARS-CoV-2, virus-like particle (VLP), antigen density, pseudotyping, variants, ACE2, neutralization, Sf9 insect cells

## Abstract

Virus-like particles (VLPs) have been proposed as an attractive tool in SARS-CoV-2 vaccine development, both as (1) a vaccine candidate with high immunogenicity and low reactogenicity and (2) a substitute for live virus in functional and neutralization assays. Though multiple SARS-CoV-2 VLP designs have already been explored in Sf9 insect cells, a key parameter ensuring VLPs are a viable platform is the VLP spike yield (i.e., spike protein content in VLP), which has largely been unreported. In this study, we show that the common strategy of producing SARS-CoV-2 VLPs by expressing spike protein in combination with the native coronavirus membrane and/or envelope protein forms VLPs, but at a critically low spike yield (~0.04–0.08 mg/L). In contrast, fusing the spike ectodomain to the influenza HA transmembrane domain and cytoplasmic tail and co-expressing M1 increased VLP spike yield to ~0.4 mg/L. More importantly, this increased yield translated to a greater VLP spike antigen density (~96 spike monomers/VLP) that more closely resembles that of native SARS-CoV-2 virus (~72–144 Spike monomers/virion). Pseudotyping further allowed for production of functional alpha (B.1.1.7), beta (B.1.351), delta (B.1.617.2), and omicron (B.1.1.529) SARS-CoV-2 VLPs that bound to the target ACE2 receptor. Finally, we demonstrated the utility of pseudotyped VLPs to test neutralizing antibody activity using a simple, acellular ELISA-based assay performed at biosafety level 1 (BSL-1). Taken together, this study highlights the advantage of pseudotyping over native SARS-CoV-2 VLP designs in achieving higher VLP spike yield and demonstrates the usefulness of pseudotyped VLPs as a surrogate for live virus in vaccine and therapeutic development against SARS-CoV-2 variants.

## 1. Introduction

COVID-19, the disease caused by the SARS-CoV-2 coronavirus, has led to a significant global burden over the last 3.5 years, totaling >760 M confirmed cases and ~7 M deaths worldwide [1]. Early development and rollout of vaccines played key roles in protecting against COVID-19 and reducing transmission of SARS-CoV-2. To date, more than 5.5 billion humans have received at least one dose of a COVID-19 vaccine, while more than 13 billion doses have been administered worldwide since late 2020 [1]. Despite the initial success of SARS-CoV-2 vaccines, primarily mRNA lipid nanoparticles (LNPs), key challenges remain for future vaccine development. Both the high mutability [2,3] and transmissibility [4,5] of SARS-CoV-2, resulting in the rapid emergence of more drifted variants [6], coupled with the waning immunity in humans previously vaccinated or infected [7,8,9], have necessitated the rollout and update of booster vaccines in efforts to reduce more severe outcomes. However, given the continual decline in booster vaccine uptake [10] and the continued circulation of the virus for the foreseeable future [1], there is a critical need to develop next-generation SARS-CoV-2 vaccines.

In addition to the need for more effective vaccines, the SARS-CoV-2 vaccine and therapeutic development also face key safety challenges. Working with SARS-CoV-2 virus requires biosafety level 3 (BSL-3) containment, severely limiting the number of experiments that can be performed due to high cost and limited availability of facilities [11]. To address this challenge, human immunodeficiency virus (HIV-1), murine leukemia virus (MLV), and vesicular stomatitis virus (VSV) pseudotyped with the SARS-CoV-2 spike protein have been most commonly used as substitutes for live virus to study binding properties and quantify neutralizing antibody titers, among other applications [12]. Despite their utility, there are several limitations for pseudovirus systems that are still replication competent, most notably safety concerns and low yields for the HIV-1 platform, morphology mismatch between VSV pseudovirus (bullet) and SARS-CoV-2 (spherical), as well as broader difficulties in quantifying pseudovirus titers and ensuring similar antigen surface density to that on the SARS-CoV-2 virus [12,13]. Taken together, there is a great need for more easily characterizable biological tools such as viral cDNA technologies [14,15], virosomes [16], and virus-like particles [17] that can emulate native SARS-CoV-2 virus.

Virus-like particles (VLPs) represent an attractive platform to serve a dual purpose in SARS-CoV-2 vaccine development, offering several unique advantages both as a vaccine candidate and as a replacement for live virus in assays. SARS-CoV-2 VLPs are commonly produced by co-expressing spike (S), envelope (E), and membrane (M) proteins in a host expression system [18,19,20,21]. They mimic the structure of the native SARS-CoV-2 virus but lack viral RNA and the ability to replicate. In contrast to the mRNA LNP technologies used for SARS-CoV-2 vaccines, VLPs have a much more favorable reactogenicity profile, in line with more traditional vaccine technologies [22]. Another advantage is the rapid production timeline (2–3 months) of VLPs [23,24,25], which can address the need for swift update of the vaccine should new variants arise. Furthermore, VLPs are highly immunogenic, and performed well as a booster in mice previously vaccinated with SARS-CoV-2 mRNA LNPs, eliciting slightly higher antibody levels with greater avidity compared to an mRNA LNP booster [26]. Finally, the molecular mimicry of VLPs to native virus makes them a useful biological tool to replace live virus in vaccine and therapeutic development, allowing for binding and neutralization assays to be carried out safely at biosafety level 1 (BSL-1) conditions.

While several host expression systems have been investigated to produce SARS-CoV-2 VLPs, including ones utilizing mammalian [17,27,28,29,30] and plant cells [31,32], the baculovirus expression vector system (BEVS) in insect cells is a particularly advantageous platform due to its ability to achieve high expression levels of recombinant proteins [18,19,21,25,33,34]. A variety of designs for SARS-CoV-2 VLPs have been produced in insect cells, with most incorporating the S protein in combination with both E and M structural proteins to form budded VLPs [18,19,21,34]. Despite the successful demonstration that expressing all three proteins can lead to the production of VLPs, it is unclear if both E and M are necessary for VLP production and how the inclusion of each of these proteins affects the overall VLP spike yield, an important parameter that is largely unreported in SARS-CoV-2 VLP studies. Moreover, though a limited number of studies [35,36] have produced VLPs towards the SARS-CoV-2 variants of interest using mammalian cells, insect cell SARS-CoV-2 VLPs to date have only incorporated the S protein from the ancestral SARS-CoV-2 strain, and VLPs based on the major circulating variants have yet to be explored.

In this study, we sought to determine the minimum requirement for native SARS-CoV-2 VLP formation in Sf9 insect cells and quantify the resulting VLP spike yield. Our data demonstrated that co-expressing S protein with either E or M protein resulted in VLP formation, but the VLP spike yield was lower compared to co-expressing all three proteins. However, regardless of the combination, all three showed very low VLP spike yields (<0.1 mg/L). To overcome this limitation, we formed pseudotyped VLPs by co-expressing an S-HA fusion protein with influenza M1, which improved the VLP spike yield approximately fivefold. This improvement further translated to VLPs with a greater antigen density (~96 S monomers/VLP), closely resembling that of native SARS-CoV-2 virus (72–144 S monomers/virion). This pseudotyping strategy also led to the successful production of VLPs for the major circulating variants, including alpha (B.1.1.7), beta (B.1.351), delta (B.1.617.2), and omicron (B.1.1.529). More importantly, the pseudotyped wild-type and variant VLP spike proteins were all shown to be functional, exhibiting differential affinities for binding with ACE2. Finally, we demonstrated the utility of VLPs to test neutralizing antibody activity in a simple, acellular ELISA assay, highlighting the usefulness of VLPs as SARS-CoV-2 virus surrogates for vaccine and therapeutic development.

## 2. Results and Discussion

### 2.1. Sf9 Insect Cells Support SE, SM, and SEM VLP Formation with Low Spike Yield

**Figure 1:**
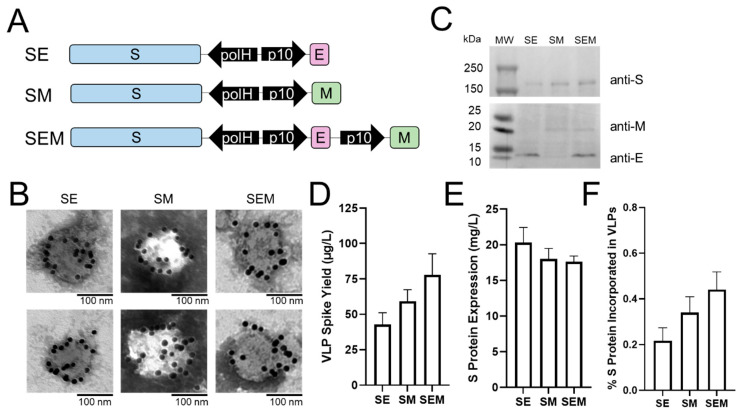
Characterizing the effects of SARS-CoV-2 structural proteins on VLP formation and spike yield. (**A**) Schematic of the recombinant baculovirus vectors used to produce SE, SM, and SEM SARS-CoV-2 VLPs in Sf9 cells. (**B**) Transmission electron microscopy (TEM) images showing anti-S immunogold-labeled VLPs. (**C**) Western blot analyses of SARS-CoV-2 proteins in VLPs. Quantification of (**D**) VLP spike yield and (**E**) cellular S protein expression level based on Western blot analyses. (**F**) % S protein incorporated in VLPs. For (**D**–**F**), data represent mean ± SE (*n* = 3, unpaired Student’s *t* test, all *p* > 0.05, not significant).

The majority of SARS-CoV-2 VLP designs reported to date have co-expressed the spike (S), envelope (E), and membrane (M) proteins in insect cells [18,19,21,34], or S, E, and M proteins, along with the nucleoprotein (N) in mammalian cells [17,27,28,29,30]. While these designs all successfully produced VLPs, it is unclear if either E or M protein on its own can support the formation of S-decorated VLPs and if the resulting VLP spike yield is increased as a result of expressing fewer number of recombinant proteins. To this end, three baculovirus vectors were generated to express S protein in combination with E and/or M proteins: SE, SM, and SEM (Figure 1A). All protein sequences were derived from the Wuhan-Hu-1 SARS-CoV-2 strain (accession #: NC_045512). Sf9 cells were then infected with the baculovirus vectors at a multiplicity of infection (MOI) of 3. At 72 h post infection, particles from the culture supernatants were harvested and processed for transmission electron microscopy (TEM) analysis (see Methods). As shown in Figure 1B, TEM analysis revealed the formation of spherical particles ~80–130 nm in diameter for all three constructs, consistent with the morphology of native SARS-CoV-2 virions (~60–140 nm diameter [37]). These particles also showed binding with multiple anti-S immunogold particles, indicating that the S protein was successfully incorporated into VLPs. Western blot analysis of these VLPs further showed that the S, E, and M proteins could be detected in the intended combinations for the SE, SM, and SEM VLPs and migrated according to their expected molecular weight (180 kDa, 12 kDa, and 25 kDa, respectively) (Figure 1C). Taken together, these data demonstrate that the formation of SARS-CoV-2 VLP in Sf9 cells does not require co-expressing S protein with both E and M proteins, and Sf9 cells support the formation of SE, SM, and SEM VLPs.

As the SE and SM VLPs require the Sf9 cells to express one less recombinant protein compared to the SEM VLP, we next examined if this leads to higher spike yields in SE and SM VLPs. The S protein in VLPs was quantified by Western blot analysis using a standard curve generated from purified S protein (Figure S1A). The SE, SM, and SEM VLP spike yields ranged from 0.04–0.08 mg/L, with SEM VLP having the highest spike yield, followed by SM and SE (Figure 1D). However, none of the yields were statistically different from each other (*p* > 0.05). Notably, the spike yields for all three native VLP constructs reported here are markedly low, particularly when compared to the influenza VLP hemagglutinin (HA) yield, which typically exceeds 1 mg/L [38]. When the S protein in cell lysates was quantified using Western blot (Figure S1B), all three VLP constructs showed high S protein expression levels (~18–20 mg/L) (Figure 1E), comparable to that of HA protein [39,40]. This indicates that the cellular expression of the S protein itself is not the cause of low VLP spike yield. Therefore, other factors severely restrict the formation of native SARS-CoV-2 VLPs in Sf9 cells. The VLP formation efficiency was evaluated using the % of S protein incorporated into VLPs, defined as the VLP spike yield divided by the cellular S protein expression level. As shown in Figure 1F, only ~0.2–0.4% of the cellular S protein expressed was incorporated into VLPs (Figure 1F). This suggests that native SARS-CoV-2 VLP formation is incredibly inefficient in Sf9 cells. It has been shown that the E and M proteins alter the secretory and glycosylation pathways in mammalian cells, resulting in the retention of S protein intracellularly [41]. Given the high influenza VLP HA yield in Sf9 cells [38], we hypothesized that a pseudotyping strategy based on influenza proteins would improve the SARS-CoV-2 VLP spike yield.

### 2.2. Pseudotyping Improves SARS-CoV-2 VLP Spike Yield

**Figure 2:**
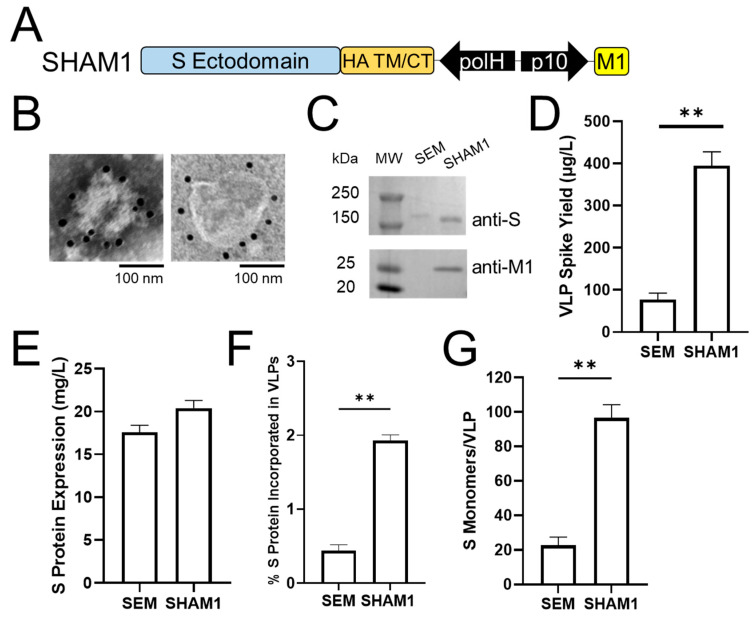
Pseudotyping improves SARS-CoV-2 VLP spike yield and antigen density. (**A**) Schematic of the recombinant baculovirus vector used to produce pseudotyped SHAM1 VLPs in Sf9 cells. The SARS-CoV-2 spike ectodomain was fused to the influenza H1N1 HA transmembrane (TM) and cytoplasmic tail (CT) domains and co-expressed with influenza M1 protein. (**B**) Transmission electron microscopy (TEM) images showing anti-S immunogold-labeled SHAM1 VLPs with a range (~100–200 nm) of diameters. (**C**) Western blot analysis of spike (top) and M1 (bottom) proteins in VLPs. Quantification of (**D**) VLP spike yield and (**E**) cellular S protein expression level based on Western blot analysis. (**F**) % S protein incorporated in VLPs. (**G**) VLP antigen density reported as the number of S monomers per VLP. For (**D**–**G**), data represent mean ± SE (*n* = 3, unpaired Student’s *t* test, ** *p* < 0.01).

Pseudotyping using influenza proteins was previously employed for SARS-CoV-1 VLPs in Sf9 cells, leading to >twofold improvement in VLP spike yield (1 mg/L) [42] compared to that of SARS-CoV-1 SEM VLPs [43]. A similar strategy was more recently utilized for SARS-CoV-2 VLPs in mammalian [44] and Sf9 insect cells [33,45], though the VLP spike yields were not reported. To determine if pseudotyping similarly improves SARS-CoV-2 VLP spike yield, a baculovirus vector was created to express the SARS-CoV-2 S ectodomain (aa 1–1213) fused to the transmembrane (TM) and cytoplasmic tail (CT) domains of an H1N1 influenza HA protein (accession #NP_040980) in combination with the influenza matrix protein M1 (denoted SHAM1, Figure 2A). For simplicity, we refer to this influenza pseudotyping strategy as “pseudotyping” throughout, including designs from other studies [44,45] that used the same HA domains but from different influenza strains. Similar to SE, SM, and SEM VLPs (Figure 1B,C), SHAM1 VLPs exhibited a spherical morphology and showed binding with multiple anti-S immunogold particles (Figure 2B). As shown in Figure 2C, Western blot analysis revealed that the S-HA and M1 proteins were incorporated into the VLPs with the correct molecular weight (S-HA, 174 kDa and M1, 25 kDa). However, the SHAM1 VLPs were slightly larger in size (~100–200 nm diameter), similar to previously engineered pseudotyped VLPs for both SARS-CoV-1 (~160 nm diameter) [42] and SARS-CoV-2 (~80–200 nm diameter) [44,45]. To examine if pseudotyping improves VLP spike yield, S protein quantification was performed as described above (Figure S1A). As expected, the SHAM1 VLP spike yield showed a significant improvement of ~fivefold to ~0.4 mg/L (Figure 2D). This yield is more comparable to the influenza VLP HA yield [38] as well as the SARS-CoV-1 VLP S-HA yield [42]. Notably, the cellular S protein expression level of SHAM1 (~20 mg/L) was not significantly different than that of SEM (Figure 2E), suggesting that the increased VLP spike yield was not a result of improved S protein expression, but was rather driven by more efficient VLP formation from influenza pseudotyped S-HA and M1 proteins. Indeed, the % of S protein incorporated into pseudotyped VLP was ~1.9%, representing a ~fivefold improvement compared to SEM VLP (Figure 2F).

Due to the higher % S protein incorporated into SHAM1 VLPs compared to native SARS-CoV-2 VLPs (Figure 1F and Figure 2F), we hypothesized that this improved S protein incorporation would result in VLPs with greater spike antigen density (i.e., the number of S monomers per VLP). Using the VLP spike yield (Figure 2D) and the total number of VLPs obtained using nanoparticle tracking analysis (see Methods), the spike antigen density of SEM VLP was determined to be ~22 S monomers/VLP (Figure 2G). In contrast, SHAM1 VLP spike antigen density was ~96 S monomers/VLP; thus, incorporating S-HA and M1 proteins into VLPs increased the antigen density by >fourfold. This improvement resulted in pseudotyped VLPs with a spike antigen density similar to that of native SARS-CoV-2 virus (~72–144 S monomers/virion [37,46,47,48]). There are several potential factors that may explain the greater antigen density of influenza pseudotyped VLPs compared to the native SARS-CoV-2 VLPs. Influenza M1 is known to be a major driving force in influenza virus budding due to its high degree of oligomerization and its strong association with the cytoplasmic tail of influenza HA at the plasma membrane [49]. In contrast to the E and M proteins, M1 is not known to alter the secretory pathway and retain other proteins. In addition, foreign transmembrane domains inserted into influenza HA have been previously shown to affect its folding into trimers and transport to the plasma membrane [50]. Future work is needed to elucidate the structural differences between the native spike and S-HA fusion proteins that might affect the conformation and in turn immunogenicity.

Despite the successful production of several pseudotyped SARS-CoV-2 VLPs using mammalian [44] and insect [33,45] expression systems, the benefit of pseudotyping in terms of vaccine efficacy is unclear. One design using HA TM/CT and M1 sequences derived from an H5N1 influenza virus resulted in SHAM1 VLPs, eliciting an inferior antibody response in mice compared to the corresponding SHA VLPs based on S-HA alone [45]. This is surprising given that M1 is known to promote influenza VLP formation in Sf9 cells [51,52], and suggests that other factors such as differences in VLP morphology or S-HA antigen density with or without M1 may explain this result. Moreover, using codon-optimized S ectodomain in these same two constructs showed the opposite effect where SHAM1 VLPs resulted in superior immunogenicity compared to SHA VLPs [45]. Further, while none of these four VLPs protected mice against lethal SARS-CoV-2 viral challenge [45], another study [44] using the same SHAM1 VLP design and codon-optimized S ectodomain did. These discrepancies observed in the immune responses elicited by pseudotyped SARS-CoV-2 VLPs highlight the need to evaluate parameters for the VLP quality, such as VLP spike yield and VLP spike antigen density described in this study. As shown in Figure 2G, different VLP designs can lead to VLPs with significantly different antigen densities, which have been correlated with antibody titer and survival against viral challenge [53,54]. In addition, the relative VLP purity may also play a role as each expression system can introduce different contaminants into the VLP sample. In the present work as well as previous studies, the baculovirus expression vector system in Sf9 cells results in the production of baculovirus vectors as well as VLPs [25]. Due to their similarities in size and density, separation of baculovirus from VLP remains challenging [55], and a well-established metric to define VLP purity is presently lacking. Therefore, including quantitative parameters in VLP characterization will help better benchmark the vaccine efficacy of different VLPs from different studies.

### 2.3. Pseudotyped Alpha, Beta, and Delta VLPs Show Higher Spike Yield Than Omicron

**Figure 3:**
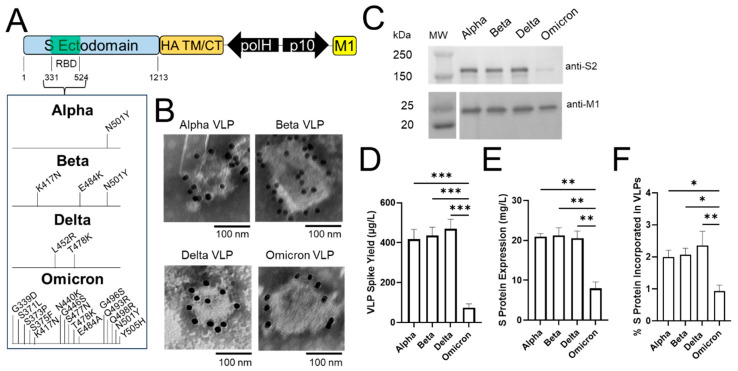
Pseudotyped alpha, beta, and delta VLPs show higher VLP spike yield than omicron. (**A**) Schematic of baculovirus vectors incorporating RBD mutations of respective variants. (**B**) Transmission electron microscopy (TEM) images showing anti-S2 immunogold-labeled VLPs for each variant. (**C**) Western blot analyses of spike (top) and M1 (bottom) proteins in VLPs. Quantification of (**D**) VLP spike yield and (**E**) cellular S protein expression level based on Western blot analyses. (**F**) % S protein incorporated in VLPs. For (**D**–**F**), data represent mean ± SE (*n* = 3, unpaired Student’s *t* test, * *p* < 0.05, ** *p* < 0.01, *** *p* < 0.001).

VLP designs in insect cells thus far have only incorporated the S protein from the ancestral SARS-CoV-2 strain, and VLPs based on the major circulating variants have yet to be explored using the baculovirus expression vector system in Sf9 cells. Given the rapid emergence of new SARS-CoV-2 variants, we next sought to produce pseudotyped VLPs for several key variants reported to date, including alpha (B.1.1.7), beta (B.1.351), delta (B.1.617.2), and omicron (B.1.1.529). These variants contain mutations in the receptor binding domain (RBD) of the S protein, ranging from as few as 1 mutation in alpha to as many as 15 mutations in the case of omicron [5]. To determine if these mutations still allow for the formation of pseudotyped VLPs in Sf9 cells, the protein sequences of the variant S RBDs were cloned in place of the Wuhan-Hu-1 (denoted as “WT”) S protein RBD using the SHAM1 baculovirus vector as backbone (Figure 3A). Following infection with respective baculovirus vectors, particles were harvested and prepared for TEM and Western blot analysis as described above (Figure 1B and Figure 2B), with one exception: anti-S2 antibody was used to ensure the same binding affinity for variant S protein detection and quantification. As shown in Figure 3B,C and Figure S2, TEM, immunogold labeling, and Western blot analysis revealed that all four variant SHAM1 VLPs exhibited a spherical morphology with a diameter ranging ~100–200 nm, showed binding with multiple anti-S2 immunogold particles, and incorporated expected proteins with the correct molecular weight (S-HA, 174 kDa and M1, 25 kDa), respectively, similar to WT SHAM1 VLPs as observed in Figure 2B,C. Therefore, the pseudotyping strategy allows for the formation of alpha, beta, delta, and omicron VLPs in Sf9 cells.

Although all four variants successfully formed pseudotyped VLPs, the S protein band for omicron was much weaker compared to the other three variants (Figure 3C). The Western blot quantification using anti-S2 antibody (Figure S3A) showed that the omicron VLP spike yield (~0.07 mg/L) was ~sixfold lower than alpha, beta, and delta (~0.4 mg/L, Figure 3D). Interestingly, the omicron S protein cellular expression level (~8 mg/L) was ~2.5-fold lower than the other three variants (~20 mg/L, Figure 3E and Figure S3B), suggesting that this reduced cellular expression was partially offsetting the benefit of pseudotyping in enhancing VLP formation. Indeed, the % of S protein incorporated into omicron VLP was ~0.9% (Figure 3F), which was not as high as the other variants but still >twofold higher than the native SARS-CoV-2 VLPs (Figure 1F).

As the omicron S RBD has 15 mutations compared to WT (Figure 3A), it is unclear which mutations or specific combinations thereof are responsible for the lower S protein expression level. One other study using Vero-E6 cells reported reduced omicron S protein expression compared to WT [56], though the specific mutation (N679K) responsible for the observed lower yield resides outside the RBD, and was not included in our VLP design. One important implication of reduced omicron S protein expression during infection or potentially mRNA vaccination may lead to a less productive antibody response against the S protein, which may in turn lead to more breakthrough infections [56]. Nevertheless, our data demonstrated that the baculovirus expression vector system in Sf9 cells is useful in evaluating and characterizing S protein expression and SARS-CoV-2 VLP formation. The resulting pseudotyped WT and variant VLPs further represent an attractive alternative to live viruses for the study of S protein binding and neutralization properties.

### 2.4. Pseudotyped VLPs Are Functional and Bind ACE2 with Varying Affinity

**Figure 4:**
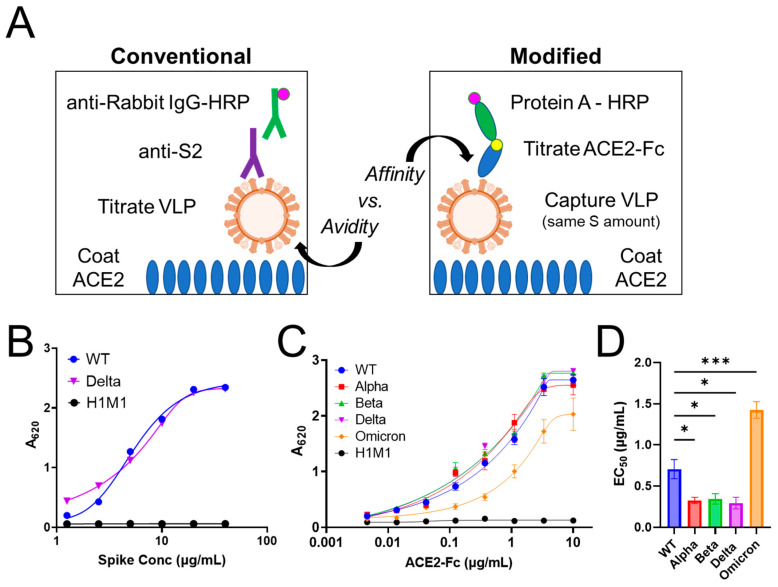
Pseudotyped VLPs are functional and show binding to ACE2 with varying affinity. (**A**) Schematic of conventional sandwich ELISA vs. a modified ELISA. Corresponding binding curve for indicated VLPs is shown in (**B**,**C**), respectively. (**D**) Quantification of the EC_50_ based on nonlinear regression of binding curve data in (**C**). For (**C**,**D**), data represent mean ± SE (*n* = 3, unpaired Student’s *t* test, * *p* < 0.05, *** *p* < 0.001).

SARS-CoV-2 viral infection is initiated by the binding of S protein to its target receptor, angiotensin-converting enzyme 2 (ACE2) [57]. ELISA has proven a useful method to rapidly assess the relative binding affinities between WT and several variant S protein RBDs to ACE2 [58,59]. However, this assessment using VLPs, which would allow for binding of S protein to ACE2 in a more physiologically relevant condition, has not been explored yet. To this end, we next sought to evaluate the relative binding affinities of pseudotyped WT, alpha, beta, delta, and omicron VLPs to ACE2 using conventional sandwich ELISA, where VLPs are titrated on ACE2-coated plates and quantified with anti-S2 followed by anti-rabbit IgG-HRP antibodies (Figure 4A, left panel). Since the higher binding affinity of the delta RBD with ACE2 compared to WT RBD is well established [58,59], these two pseudotyped VLPs were tested first. As shown in Figure 4B, compared to the negative control influenza H1M1 VLPs, both WT and delta VLPs showed a dose-dependent response, indicating that the S protein in both VLPs is functional and can bind ACE2. However, there was no difference observed in the EC_50_ (half maximal effective concentration) between WT and delta VLPs, in contrast to previous studies using soluble RBDs [58,59]. A plausible explanation for this discrepancy is that, in the conventional sandwich ELISA, the interaction between ACE2 and S protein on VLPs is influenced by avidity (Figure 4A, left panel). Avidity effects have been shown to enhance the binding affinity >10–1000-fold [60,61], and may mask binding affinity differences depending on the experimental setup [62,63]. Notably, the delta VLPs showed stronger binding than the WT VLPs, but this was evident only at lower concentrations of VLPs (≤2.5 µg/mL S protein) (Figure 4B). This observation further supports avidity effects on binding behavior in this conventional sandwich ELISA setup.

To eliminate the influence of the avidity effect, we modified the ELISA as depicted in the right panel of Figure 4A. In this modified format, ACE2-Fc protein is titrated in wells containing the same amount of captured VLPs, which allows for the assessment of the monovalent interaction between S protein on VLPs and ACE2-Fc. A critical step here is ensuring that the same amount of VLPs based on S protein amount are captured in each well. To achieve this, 10 µg/mL of VLPs (S protein) was loaded, and the captured VLPs were quantified using anti-S2 followed by anti-rabbit IgG-HRP antibodies. All wells showed the same colorimetric signal (Figure S4), confirming that they contained the same amount of S protein on captured VLPs.

Once the same amount of captured VLPs was confirmed, the modified ELISA was performed by titrating ACE2-Fc and detecting with protein A-HRP (Figure 4A, right panel). All five pseudotyped SARS-CoV-2 VLPs showed a dose-dependent response, indicating that the S protein in all VLPs is functional and can bind ACE2-Fc (Figure 4C). Based on the binding curves, EC_50_ values were determined for each of the S:ACE2-Fc interactions. Compared to WT S:ACE2-Fc (EC_50_ 0.7 µg/mL), the delta S:ACE2-Fc EC_50_ value was ~twofold lower (~0.3 µg/mL), indicating a stronger binding affinity between the delta VLP S protein and ACE2-Fc (Figure 4D). Beta behaved similarly to delta. In contrast, the binding of omicron to ACE2-Fc was significantly weaker than WT, with ~twofold greater EC_50._ These data agree with previous studies using soluble RBDs [58,59], confirming the validity of the modified ELISA format for measuring the relative binding affinity. Additionally, our data revealed that the binding of alpha to ACE2-Fc was similar to beta. Interestingly, this result suggests that the N501Y mutation in S protein, common to both alpha and beta, drives the increase in binding affinity to ACE2 compared to WT. Despite also sharing the N501Y mutation, omicron showed much weaker binding affinity than WT. A computational modeling study suggested that nine other mutations in the omicron RBD would decrease its binding affinity to ACE2 [64]. The weaker binding of omicron S protein to ACE2 implicates other interactions such as omicron’s preference for cathepsin L instead of TMPRSS2 as the driving force behind its enhanced infectivity and transmissibility [65].

### 2.5. Using Pseudotyped VLPs as Surrogates for Live Virus in a Neutralization Assay

**Figure 5:**
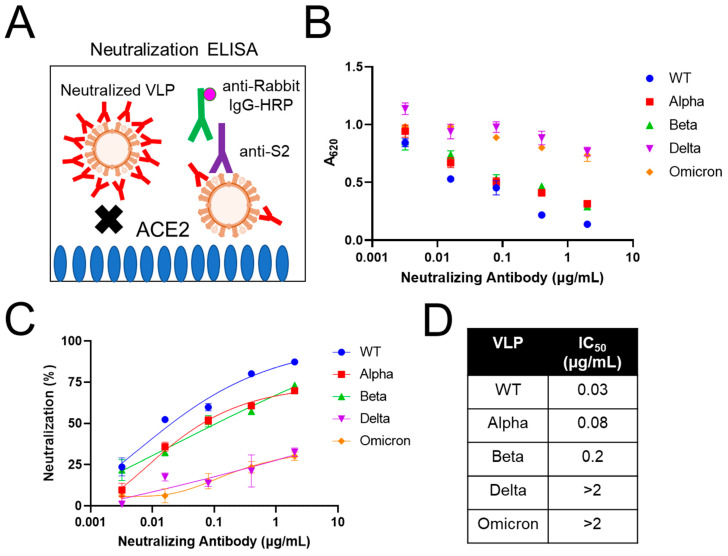
Using pseudotpyed VLPs as SARS-CoV-2 virus surrogates to measure antibody neutralization activity. (**A**) Schematic showing neutralization ELISA setup. VLPs were preincubated with a neutralizing antibody to prevent capture by ACE2. (**B**) Signal reduction indicating neutralization of indicated VLPs. (**C**) The percent neutralization was determined by comparing the signal from VLPs preincubated with or without neutralizing antibody. (**D**) IC_50_ calculated from data in (**C**) using nonlinear regression analysis. For (**B**,**C**), data represent mean ± SE (*n* = 3).

In addition to understanding virus binding properties, another important aspect of SARS-CoV-2 vaccine and therapeutic development is the screening of viral inhibitors and antibodies, particularly for their virus-neutralizing properties. However, neutralization assays remain challenging due to the BSL-3 requirement for experiments involving live SARS-CoV-2 virus [66]. As a result, pseudoviruses [67,68,69] or VLPs [21,35,36,70] have been used as virus surrogates in neutralization assays. In these studies, a reporter system (e.g., luciferase or GFP) is incorporated in the pseudovirus or VLP to evaluate if antibodies can block their ability to enter the target cell (e.g., ACE2-expressing cells). In the present work, we developed a simple, acellular neutralization assay based on ELISA (Figure 5A), allowing us to measure antibody neutralization activity against VLPs without relying on a reporter system. In the context of this assay, neutralization is defined as the ability of the neutralizing antibody to block the binding of VLPs to ACE2 [71,72]. Briefly, 5 µg/mL of VLPs was preincubated with varying concentrations of a neutralizing monoclonal antibody (mAb) raised against WT S protein, and then loaded into ACE2-coated wells. After washing away unbound VLPs, captured VLPs were detected with anti-S2 followed by anti-rabbit IgG-HRP antibodies. As shown in Figure 5B, increasing concentration of neutralizing mAb prevented more VLPs from being captured by ACE2, leading to a reduction in signal. To quantify the percent of neutralization, the signal for a given neutralizing mAb concentration was normalized against the signal for VLPs preincubated without neutralizing mAb. Percent neutralization was then plotted as a function of mAb concentration (Figure 5C), and the half maximal inhibitory concentration (IC_50_) was determined to compare the antibody neutralization against both WT and variant VLPs (Figure 5D).

Overall, the neutralizing mAb blocked WT VLP binding to ACE2 most effectively, reaching >87% neutralization (Figure 5C), with the lowest IC_50_ value (0.03 µg/mL) among all VLPs tested (Figure 5D). This is expected, as the mAb tested in this study was raised against the WT S protein. Comparatively, the neutralizing mAb blocked the alpha and beta VLPs less effectively, with IC_50_ values ~2.7- and ~6.7-fold higher than that of the WT VLP, respectively (Figure 5D). This result indicates that in addition to the N501Y mutation, which is shared between alpha and beta variants, the E484K and/or K417N mutations also affect the neutralization activity of this mAb. For delta and omicron VLPs, <30% neutralization was observed at the highest concentration of neutralizing mAb tested (Figure 5C), and the IC_50_ values could not be determined (Figure 5D). This result suggests that the L452R and/or T478K mutations found in the delta variant nearly abolished the neutralization activity of the mAb tested. These results are consistent with the data from the manufacturer, which showed significantly less neutralization of delta and omicron pseudoviruses compared to WT in a cell-based microneutralization assay. Therefore, the ELISA-based acellular neutralization assay developed here can provide quantitative data on the efficacy of neutralizing antibodies. It is important to note that this assay evaluates the ability of neutralizing antibodies or inhibitors to block binding of VLPs to ACE2. Other cellular-based assays are needed to further demonstrate neutralization through the point of cellular fusion and entry. Nevertheless, combined with the advantages of its fast completion in less than a day and adaptability for high-throughput screening of antibodies and viral inhibitors, this assay adds another effective approach to the neutralization assay toolkit.

In summary, pseudotyped VLPs produced in Sf9 insect cells are a promising dual-purpose platform in the fight against COVID-19. Compared to the critically low VLP spike yields of native SE, SM, and SEM VLPs produced in Sf9 insect cells, influenza pseudotyped VLP spike yields were significantly improved, resulting in VLPs with antigen density similar to that of the native SARS-CoV-2 virus. We successfully employed this pseudotyping strategy to produce VLPs incorporating the alpha, beta, delta, and omicron RBDs, the first example of variant VLPs produced in Sf9 insect cells. Despite the lower omicron VLP spike yield, we were able to demonstrate the functionality of all pseudotyped VLPs by quantifying their differential binding affinity to ACE2. Finally, we showed the utility of pseudotyped VLPs as virus surrogates in evaluating neutralizing antibody activity in a simple, acellular ELISA format. Taken together, pseudotyped VLPs produced in Sf9 cells represent a safe and effective tool that allows for the investigation of SARS-CoV-2 viral binding properties and antibody neutralization activity to be performed at BSL-1 facilities. This accessibility opens avenues for the wider research community to contribute to the collective endeavor in combatting COVID-19.

## 3. Methods

### 3.1. Strains, Media, and Reagents

Sf9 insect cells (CRL-1711, ATCC, Manassas, VA, USA) were grown in Insect XPRESS Media (Lonza, Walkersville, MD, USA) supplemented with 10 mg/L gentamycin at 27 °C and 135 rpm agitation. DH10Bac cells (Bac-to-Bac Baculovirus Expression Systems, Life Technologies, Foster City, CA, USA) were grown in Luria–Bertani (LB) medium containing 50 µg/mL kanamycin, 7 µg/mL gentamicin, and 10 µg/mL tetracycline. Unless otherwise stated, all media and antibiotics were purchased from Thermo Fisher Scientific (Waltham, MA, USA) and all other chemicals were purchased from Sigma-Aldrich (St. Louis, MO, USA). All primers were purchased from Integrated DNA Technologies (Coralville, IA, USA).

### 3.2. Recombinant Baculovirus Generation

The DNA sequences encoding the SARS-CoV-2 S, E, and M proteins were amplified from gBlock fragments purchased from Integrated DNA Technologies. First, the S gene was cloned into the *BamHI/HindIII* site in plasmid pFastBac Dual to create the intermediary plasmid pFastBac Dual-S. E and M genes were cloned into the *XhoI/XmaI* site in separate pFastBacDual-S plasmids to create plasmids pFastBacDual-SE and pFastBacDual-SM, respectively. The expression cassette for M including the p10 promoter, M gene, and HSV terminator was then PCR amplified from pFastBac Dual-SM and cloned into the *AvrII* site of pFastBac Dual-SE to create pFastBac Dual-SEM.

Previously, influenza HA and M1 genes were cloned into the *XbaI/HindIII* and *KpnI/XmaI* site in plasmid pFastBac Dual, respectively, to create plasmid pFastBac Dual-H1M1 [73]. This plasmid served as the backbone for all pseudotyped SARS-CoV-2 S/Influenza HA fusion plasmids. The S/HA fusion fragment was created using overlap extension PCR. First, the S ectodomain fragment (S-ECTO) and HA transmembrane and cytoplasmic tail domain fragment (HA_TMCT) were amplified from pFastBac Dual-S and pFastBac Dual-H1M1, respectively. S-ECTO and HA-TMCT fragments were then spliced and cloned into the *XbaI/HindIII* site of pFastBac Dual-H1M1, replacing the full-length HA gene to create pFastBac Dual-SHAM1.

Receptor binding domain (RBD) mutations for the SARS-CoV-2 alpha (N501Y), beta (K417N, E484K, N501Y), and delta (L452R, T478K) variants were introduced into separate pFastBac Dual-SHAM1 plasmids using Quikchange mutagenesis [74] to create pFastBac Dual-SHAM1-Alpha, pFastBac Dual-SHAM1-Beta, and pFastBac Dual-SHAM1-Delta. The sequence encoding the first 685 amino acids of the S ectodomain including the omicron RBD sequence was amplified from a gBlock gene fragment and cloned into the *XbaI/ApaI* site in pFastBac Dual-SHAM1 plasmid to create pFastBac Dual-SHAM1-Omicron. The templates and primers used for all PCR reactions are listed in Table S1. All DNA sequences were confirmed using Sanger sequencing. 

The recombinant baculovirus genome (i.e., bacmid) was created by transforming each pFastBac Dual plasmid into DH10Bac via transposition. After confirming the recombination events using blue/white colony screening and PCR, the recombinant bacmids were purified using a PureLink HiPure Plasmid Miniprep kit (Invitrogen, Carlsbad, CA, USA). The purified bacmids were then transfected into Sf9 cells using Cellfectin II (Invitrogen) according to manufacturer’s protocol to generate recombinant baculovirus P1 stocks, which were amplified in Sf9 cells to obtain high-titer P2 baculovirus stocks for use in protein expression and VLP production experiments.

### 3.3. Cellular Expression and Protein Quantification

Cellular expression of S, E, and M proteins as well as S/HA and M1 was confirmed using Western blot analysis of cell lysates using anti-S (40591-T62, Sino Biological US Inc., Wayne, PA, USA), anti-M (NBP3-07058, Novus Biologicals, Centennial, CO, USA), anti-E (NBP3-07060, Novus Biologicals), and anti-M1 (PA532253, Invitrogen). For variant S proteins, anti-S2 (40590-T62, Sino Biological US Inc.) was used, as the S2 domain was conserved across all variants. To quantify cellular yields of all S protein constructs, S protein standard (40589-V08B1, Sino Biological US Inc.) was used. Following primary antibody staining, alkaline phosphatase-conjugated anti-mouse or anti-rabbit IgG secondary antibody (Life Technologies) was used. NBT-BCIP (Thermo Fisher Scientific) was used to develop Western blots. Densitometric analysis of Western blots was performed using a Gel Doc EZ™ Imager (Bio-Rad, Hercules, CA, USA) to generate standard curves for S, which were then used to calculate the S cellular expression level.

### 3.4. Virus-like Particle (VLP) Production and Characterization

Influenza VLPs were produced from Sf9 cells infected at an MOI of 3 and harvested 72 hpi. Cell debris was removed from the supernatant by centrifugation at 300× *g* for 20 min followed by 10,000× *g* for 20 min. The cleared supernatant was ultracentrifuged at 150,000× *g* for 2 h, and the pellet containing VLPs was resuspended in PBS containing 40% glycerol. All centrifugation steps were carried out at 4 °C.

The number of VLPs was quantified using a NanoSight NS300 particle tracking system (Malvern Panalytical, Malvern, UK). Specifically, VLPs were diluted in PBS to manufacturer’s recommended concentrations prior to injection. Videos of 60 s in length were recorded for each sample, and the particle concentration was determined using the nanoparticle tracking analysis (NTA) software provided with the NS300 system. The amount of S in each VLP preparation was quantified by densitometric analysis of Western blots as described in the section above. VLP spike yield (defined as the amount of S protein in VLPs), was determined by densitometric analysis of Western blots as described above. For each VLP construct, the particle concentration and VLP spike yield are represented as the mean of three independent experiments. The spike antigen density (defined as the number of S monomers per VLP) was then determined by using the equation below:

(1) $$Spike\;Antigen\;Density=\frac{\left(VLP\;Spike\;Yield)(\frac{1}{{MW}_{Spike}})(N_{A}\right)}{Particle\;concentration}$$

The MW_Spike_ is the molecular weight of the spike protein (180 kDa for native S protein, 174 kDa for S-HA fusion protein). N_A_ is Avogadro’s number. All appropriate conversion factors were used to calculate the antigen density in units of S monomers per VLP.

VLPs were characterized by immunogold labeling analysis using transmission electron microscopy (TEM). Briefly, VLPs were absorbed on Ni grids (Electron Microscopy Sciences, Hatfield, PA, USA) and incubated with 20 ng/µL anti-S antibody for 1 h, followed by labeling with protein G—gold nanoparticle (15 nm)—conjugates (Electron Microscopy Sciences) at a concentration of 10^11^ gold nanoparticles/mL for 30 min. Grids were stained with 2% phosphotungstic acid (PTA) and allowed to dry 1 h prior to TEM analysis on a JEM-1400 Transmission Electron Microscope, 80 kV (JEOL, Peabody, MA, USA).

### 3.5. VLP Binding and Neutralization ELISA

VLP binding to ACE2 was analyzed using ELISA. 96-well MaxiSorp plates (BioLegend, San Diego, CA, USA) were coated with 2 µg/mL of recombinant human ACE2 (carrier free) (BioLegend) overnight at 4 °C. After washing 3× with ELISA wash buffer, wells were blocked with a 1× ELISA diluent (5× stock, Thermo Fisher Scientific) for 2 h at room temperature. VLPs containing 10 µg/mL S protein/well in 1× ELISA diluent were captured for 2 h at room temperature. After washing 3×, recombinant protein ACE2-Fc (BioLegend) was titrated from 0.004–10 µg/mL well for 2 h at room temperature. After washing 3× to remove unbound ACE2-Fc, detection was performed using HRP protein A (1:1000, BioLegend). After 5× washing, visualization was performed using TMB substrate (BioLegend). Absorbance was read at 620 nm using a SpectraMax M5 plate reader. EC_50_ values were calculated with GraphPad Prism, fitting to a five-parameter logistic curve.

To confirm that an equivalent amount of VLPs were captured for each sample in the above experiment, three VLP-captured wells for each sample were labeled with anti-S2 (1:1000, Sino Biological US Inc.), washed 3×, and detected with anti-rabbit IgG-HRP (1:2000, PI31460, Thermo Fisher Scientific) (Figure S4). Five times washing, visualization with TMB, and absorbance readings were performed as described above.

Neutralization of VLPs was investigated using a similar ELISA setup. First, VLPs containing 5 µg/mL S were incubated with 0.0032–2 µg/mL SARS-CoV-2 (2019-nCoV) Spike-Neutralizing Monoclonal Antibody (40591-MM48, Sino Biological US Inc.) overnight at 4 °C. On the same day, plates were coated with 2 µg/mL ACE2/well as described above and stored overnight at 4 °C. The next day, preincubated VLPs were then loaded into the ACE2-coated wells for 2 h. After washing 3× to remove unbound (i.e., neutralized) VLPs, captured VLPs were labeled with anti-S2, detected with anti-rabbit IgG-HRP (1:2000), and visualized with TMB as described above. Percent neutralization of VLPs was calculated as the difference in A_620_ signal for VLPs preincubated with and without neutralizing antibody divided by the A_620_ signal for VLPs preincubated without neutralizing antibody. Following nonlinear fitting, IC_50_ values were calculated with GraphPad Prism.

### 3.6. Statistical Analysis

Statistical analysis was performed using unpaired Student’s *t* test. All data are represented as the mean of three independent experiments and error bars represent the standard error of mean (SE). * *p <* 0.05, ** p < 0.01, *** p < 0.001; not significant *p >* 0.05.

## Data Availability

Data are contained within this article.

## Supplementary Materials

The supporting information can be downloaded at: https://www.mdpi.com/article/10.3390/ijms241914622/s1.
