# Supplementary material for: Pseudotyping Improves the Yield of Functional SARS-CoV-2 Virus-like Particles (VLPs) as Tools for Vaccine and Therapeutic Development
Source: Int J Mol Sci. 2023 Sep 27;24(19):14622. doi: 10.3390/ijms241914622 (PMC10572262; doi:10.3390/ijms241914622)
Supplement: Supplementary file 1 [file ijms-24-14622-s001.zip › ijms-2585157-supplementary.pdf]

Article

Pseudotyping Improves the Yield of Functional SARS-CoV-2 Virus-like Particles (VLPs) as Tools for Vaccine and Therapeutic Development

Andrew J. Zak, Trang Hoang, Christine M. Yee, Syed M. Rizvi, Ponnandy Prabhu and Fei Wen *

Department of Chemical Engineering, University of Michigan, Ann Arbor, MI 48109, USA; prabhup@umich.edu (P.P.)
* Correspondence: feiwenum@umich.edu

Table S1. Templates and primers used in this study.

Template	PCR product	Primer name	Sequence
A/PR/8/34 H1N1 cDNA	HA	HA-For	AATCTAGAATGAAGGCAAACCTACTGGTCC
		HA-Rev	ACAAGCTTTCAGATGCATATTCTGCACTGC
	M1	M1-For	TCCCGGTACCTCACTTGAACCGTTGC
		M1-Rev	ATCACCCGGGATGAGTCTTCTAACC
gBlock S	S	S-For	CGGGCGCGGATCCATGTTTGTGTTCCTTGTTCTT CTTC
		S-Rev	ACTTCTCGACAAGCTTTTAAGTGTAGTGCAGCTT CACGCCCTTCA
gBlock E	E	E-For	ACCATGGCTCGAGTTACACAAGCAAGTCTGG
		E-Rev	TGATCACCCGGGATGTATTCGTTTGTTTCAGAAG AAACA
gBlock M	M	M-For	ACCATGGCTCGAGTTATTGTACAAGGAGGGCG
		M-Rev	TGATCACCCGGGATGGCTGACTCAAATGGAAC
pFastBac Dual SM	M cassette	Mcas-For	TTGAGCCTAGGGTGGCTATGGCAGGGCTTGC
		Mcas-Rev	GATCTCCTAGGATACGGACCTTTAATTCAACCCA AC
gBlock – S Omicron	S Omicron	Om-Rev	TGTGTAAGCGATAATTGACTGGGACGCTAC
**Template**	**Quikchange Primer**		**Sequence**
pFastBac Dual SHAM1	K417N-For		GCCCCAGGACAAACAGGTAACATAGCTGACTAT AACTAC
	K417N-Rev		CTATGTTACCTGTTTGTCCTGGGGCAATCTGTCT G
	L452R-For		GTGGGAGGCAACTACAACTACAGATACAGACTT TTTAGG
	L452R-Rev		CTGTATCTGTAGTTGTAGTTGCCTCCCACCTTGCT ATC
	T478K-For		AAGCCGGTAGCAAGCCATGTAATGGCGTCGAGG G
	T478K-Rev		CGCCATTACATGGCTTGCTACCGGCTTGATAGAT TTCCG
	E484K-For		AATGGCGTCAAGGGATTTAATTGTTATTTCCCGT TGCAATCATAC
	E484K-Rev		AATTAAATCCCTTGACGCCATTACATGGCGTGCT ACCG
	N501Y-For		TTTCAGCCGACGTACGGCGTTGGTTATCAACCG
	N501Y-Rev		ATAACCAACGCCGTACGTCGGCTGAAAGCCGTA TGATTGC

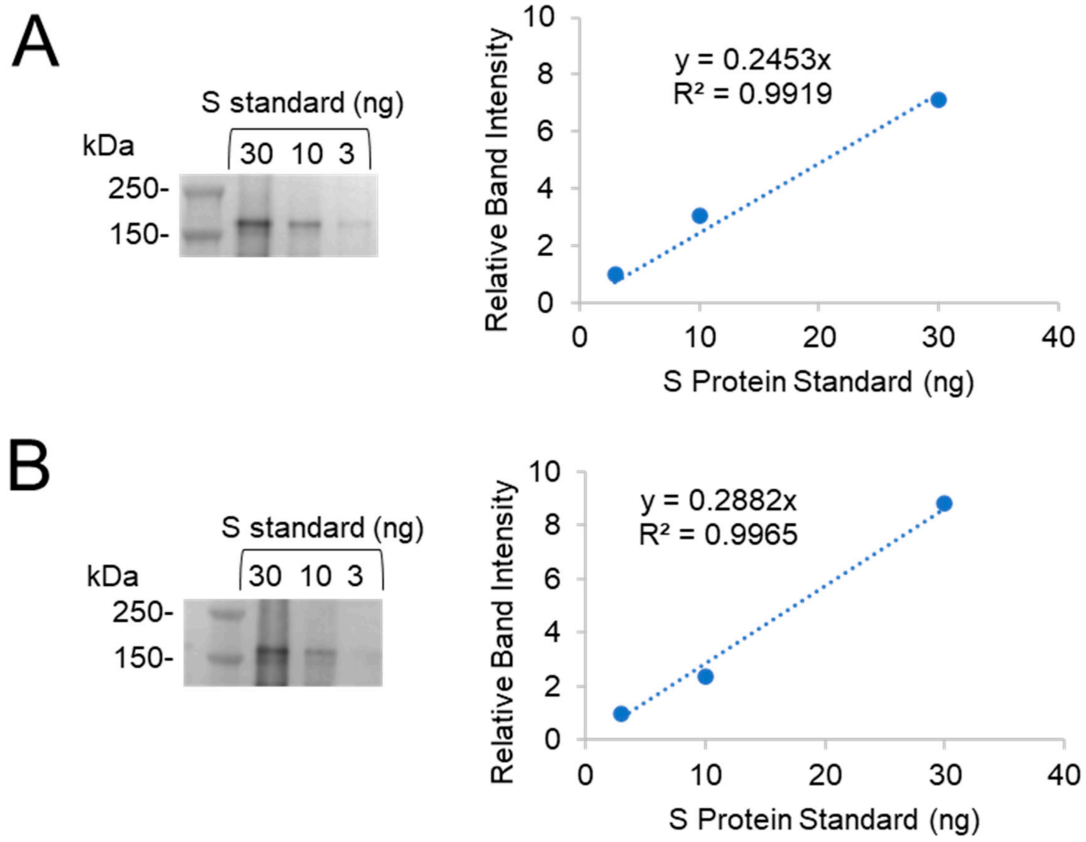

Figure S1. Standard curves for Western blot quantification of S protein in SE, SM, SEM and WT SHAM1 (A) VLPs and (B) cell lysates.

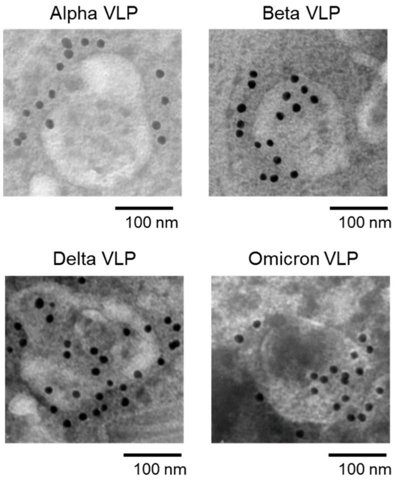

Figure S2. Representative TEM images of pseudotyped variant VLPs in the 150-200 nm size range. Immunogold-labeling was performed using anti-S2.

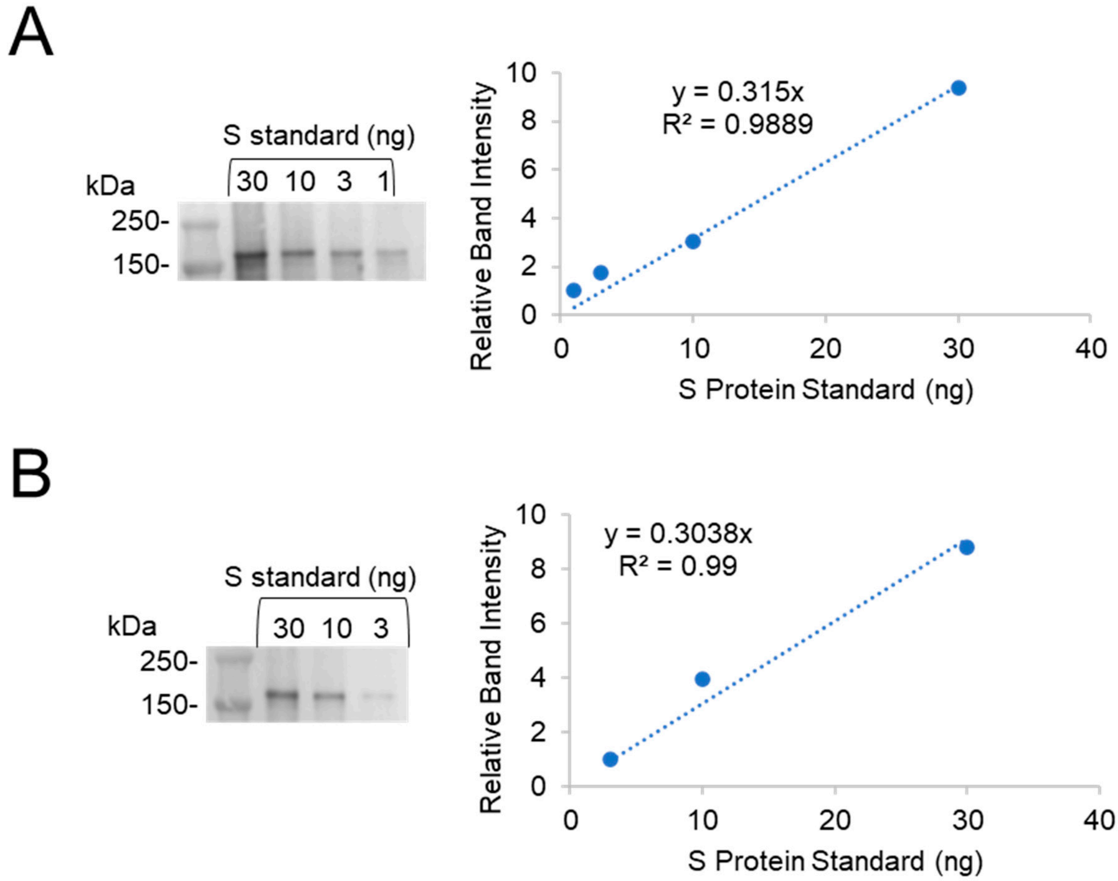

Figure S3. Standard curves for Western blot quantification of S protein in pseudotyped variant (A) VLPs and (B) cell lysates using anti-S2.

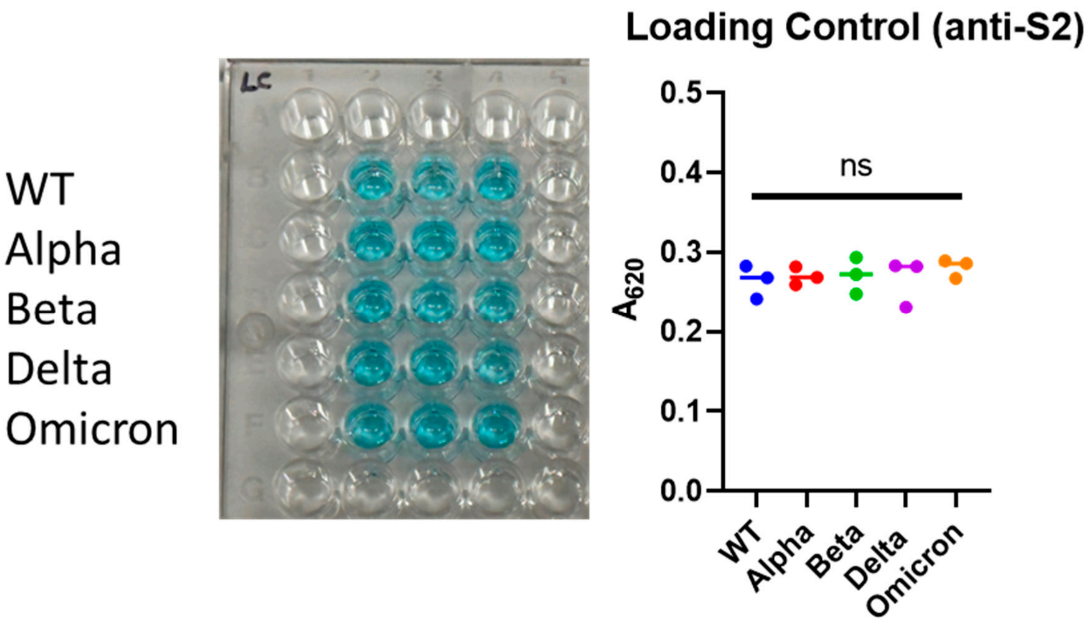

Figure S4. Loading controls to ensure same quantity of S variant VLPs are captured on the plate.

Data represent mean ± SE (n = 3, unpaired Student's t test, all $p > 0.05$, not significant).
